# Efficacy of Pelvic Floor Muscle Training for Postoperative Patients With Rectal Cancer: A Systematic Review and Meta-Analysis

**DOI:** 10.7759/cureus.50287

**Published:** 2023-12-10

**Authors:** Yuki Nakashima, Kenichi Fudeyasu, Yuki Kataoka, Shunsuke Taito, Takashi Ariie, Yukio Mikami

**Affiliations:** 1 Division of Rehabilitation, Department of Clinical Practice and Support, Hiroshima University Hospital, Hiroshima, JPN; 2 Department of Systematic Reviewers, Scientific Research WorkS Peer Support Group (SRWS-PSG), Osaka, JPN; 3 Department of Internal Medicine, Kyoto Min-iren Asukai Hospital, Kyoto, JPN; 4 Section of Clinical Epidemiology, Department of Community Medicine, Kyoto University Graduate School of Medicine, Kyoto, JPN; 5 Department of Healthcare Epidemiology, School of Public Health, Kyoto University Graduate School of Medicine, Kyoto, JPN; 6 Department of Physical Therapy, School of Health Sciences at Fukuoka, International University of Health and Welfare, Fukuoka, JPN; 7 Department of Rehabilitation Medicine, Hiroshima University Hospital, Hiroshima, JPN

**Keywords:** colorectal cancer, fecal incontinence, rehabilitation, pelvic floor muscle training, rectal cancer

## Abstract

This study aimed to assess the effectiveness of pelvic floor muscle training (PFMT) for fecal incontinence (FI) and health-related quality of life (HR-QOL) after colorectal cancer surgery. This systematic review (SR) and meta-analysis included randomized controlled trials (RCTs) that examined the effects of PFMT after colorectal cancer surgery, which were extracted from several databases in January 2023. The primary outcomes were FI (Wexner scores), HR-QOL, and adverse events. We used the Grading of Recommendations, Assessment, Development, and Evaluation (GRADE) approach to assess the certainty of evidence (CoE). A total of seven RCTs were included. Our SR results suggested that PFMT showed little to no difference in FI (mean difference 0.62 higher; 95% CI: -1.26 to 2.5, low CoE) and adverse events (risk ratio 5.78; 95% CI: 0.28-117.22, low CoE). Two adverse events occurred in the PFMT group (anastomotic stenosis, suboptimal use of laxatives) and were not observed in controls. HR-QOL was measured in two RCTs using 12-item Short Form Survey (SF-12) and fecal incontinence quality of life (FIQL). Two RCTs found no trend toward a positive impact on HR-QOL. Higher quality RCTs on colorectal cancer after surgery are required. Furthermore, extending the duration of the PFMT intervention may be necessary to ensure its success.

## Introduction and background

Fecal incontinence (FI) frequently occurs following colorectal cancer surgery. A recent systematic review (SR) reported a 24.1% prevalence of liquid FI and 6.9% of solid FI [1]. Moreover, postoperative FI in colorectal cancer is associated with the patient’s quality of life (QOL) [2]. Therefore, addressing FI after colorectal cancer surgery is important.

Pelvic floor muscle training (PFMT) is used as a conservative treatment for urinary incontinence and FI [3,4], particularly in antenatal and postnatal women [3]. Furthermore, according to a recent SR of post-colorectal cancer surgery, it may also be effective for FI [4]. However, this SR did not include randomized controlled trials (RCTs), and no meta-analyses have been conducted [4].

Several recent RCTs of PFMT for FI in postoperative patients with colorectal cancer have been reported [5,6]. A recent RCT has shown that it may be effective for bowel symptoms at six months but not at 12 months [5]. Another RCT showed significant improvement in patients with baseline Wexner scores of ≤16 points [6]. Thus, SRs involving these RCTs provide a more reliable way of demonstrating the efficacy of PFMT. Therefore, this study aimed to investigate the effect of PFMT on improving outcomes, such as FI, QOL, and adverse events after colorectal cancer surgery, through an SR and meta-analysis of RCTs.

## Review

Methods

We followed the Preferred Reporting Items for Systematic Reviews and Meta-Analyses 2020 (PRISMA-2020) guidelines for preparing this protocol (https://osf.io/2pmy8) [7]. The study protocol was registered on the Open Science Framework (https://osf.io/kqr4n/).

The inclusion criteria for the reviewed articles were RCTs that assessed individual randomization without any language or country restrictions and incorporated all forms of literature, including published and unpublished articles. Furthermore, studies were not excluded based on the observation period or publication year.

We included adult patients who had undergone surgery for colorectal cancer. Studies were included regardless of the intervention setting (in-hospital or out-of-hospital). Studies involving mixed populations where a percentage of participants were only non-surgically treated or children (<18 years) were excluded, and results for these populations were presented separately.

Exercises targeting the pelvic floor muscle groups, such as the external anal sphincter, were considered as PFMT [4]. We included studies on the effects of PFMT in postoperative patients with colorectal cancer, with no restrictions on when the exercises were performed. Additionally, we included studies that examined PFMT interventions alone or in combination with patient education, biofeedback, electrostimulation, or rectal balloons. However, we excluded studies that used electrostimulation, rectal balloons, or biofeedback alone. No treatment, usual care, or usual rehabilitation were used as controls. Other treatments (e.g., patient education, biofeedback, electrostimulation, or rectal balloon) were included as control, if the same treatments were applied to intervention groups. However, we excluded studies where the control groups received PFMT.

We defined our primary outcomes as FI, HR-QOL, and adverse events. We extracted the total Wexner score (Cleveland Clinic Florida Fecal Incontinence Scale) as a measure of FI [3]. We extracted validated disease-specific and overall health-related quality of life (HR-QOL) questionnaires (European Organization for Research and Treatment of Cancer (EORTC) QLQ-C30, EORTC QLQ-CR29, EURO-QoL 5D, SF-36, SF-12, etc.) as measures of HR-QOL. If sub-items such as physical component score and mental component score were clear, surveys were conducted, respectively. The time points for these outcome assessments were the longest point during the period of one to 12 months from the start of the intervention.

We defined our secondary outcomes as bowel dysfunction after surgery. We extracted the total low anterior resection syndrome (LARS) score as a measure of bowel dysfunction after surgery [8]. The longest point during the period of one to 12 months from the start of the intervention was extracted.

Search Method

We searched the Cochrane Central Register of Controlled Trials (CENTRAL) via Cochrane Library, MEDLINE via PubMed, Excerpta Medica Database (EMBASE) via Dialog, Physiotherapy Evidence Database (PEDro), Cumulative Index to Nursing and Allied Health Literature (CINAHL), WHO International Clinical Trials Registry Platform (WHO ICTRP) through their dedicated search portal, and ClinicalTrials.gov between January 12, 2023 and January 19, 2023 (https://osf.io/hevk2). We checked the reference lists of studies, including international guidelines [9-12], as well as the reference lists of eligible studies and articles citing eligible studies. We asked the authors of original studies for unpublished or additional data.

Data Collection and Analysis

Selection of the studies: Two independent reviewers (YN and KF) screened the titles and abstracts, followed by the assessment of eligibility based on the full texts. We contacted the original authors in case of the missing relevant data. Discrepancies were discussed by the two reviewers until they reached a consensus; otherwise, a third reviewer (TA) was consulted.

Data extraction and management: Two reviewers (YN and KF) independently performed data extraction on the included studies using a standardized data collection form. The form included author information, number of participants, types of surgery, stoma, intervention, controls, and outcomes. Discrepancies were discussed by the two reviewers until they reached a consensus or by discussion with a third reviewer (TA).

Assessment of risk of bias in included studies: The two reviewers (YN and KF) evaluated the risk of bias independently using the Risk of Bias 2 tool [13]. Disagreements between the two reviewers were resolved by discussion with a third reviewer (TA) acting as an arbiter.

Measures of treatment effects: Relative risk ratios (RRs) and 95% confidence intervals (CIs) for binary variables were pooled for the presence of adverse events. The mean or standardized mean differences and the 95% CIs were pooled for the continuous variables (i.e., FI, HR-QOL, and bowel dysfunction after surgery) and summarized based on the definition in the original article.

Handling of missing data: We requested the original authors to share data that were not presented in the published studies. The intention-to-treat (ITT) analysis was performed for all dichotomous data. For continuous data, missing data were not imputed based on the recommendation in the Cochrane Handbook [14]. We converted data from available data according to the methods of the Cochrane Handbook [14].

Assessment of heterogeneity: The statistical heterogeneity was evaluated through visual inspection of the forest plots and calculating the I^2^ statistic. When there was substantial heterogeneity (I^2^ > 50%), the reason for the heterogeneity was assessed. A Cochrane chi-squared test (Q-test) was performed for the I^2^ statistic, and statistical significance was set at P < 0.10 [15].

Assessment of reporting bias: Furthermore, we searched the clinical trial registry system (ClinicalTrials.gov and ICTRP) to explore registered but unpublished trials. The potential publication bias was assessed through visual inspection of the funnel plot; however, we did not perform an Egger test due to the limited sample size.

Meta-analysis: The meta-analysis was conducted using the Review Manager software (RevMan 5.4) and employing a random-effects model.

Summary of findings table: According to the Cochrane Handbook [14], a table showing the summary of findings was created for FI, HR-QOL, adverse events, and bowel dysfunction after surgery [15]. Corresponding risks were adopted from the median of the included trials. Furthermore, the quality of the evidence was evaluated based on the GRADE (Grading of Recommendations Assessment, Development, and Evaluation) approach for each summary of findings.

Difference Between Protocol and Review

Due to insufficient data, we were unable to perform planned subgroup analyses for the following variables: age (<65 vs. >65 years) and concomitant treatment (PFMT only vs. PFMT with concomitant therapy). In addition, we were unable to conduct the intended sensitivity analyses for the primary outcomes: exclusion of studies.

Results

**Figure 1:**
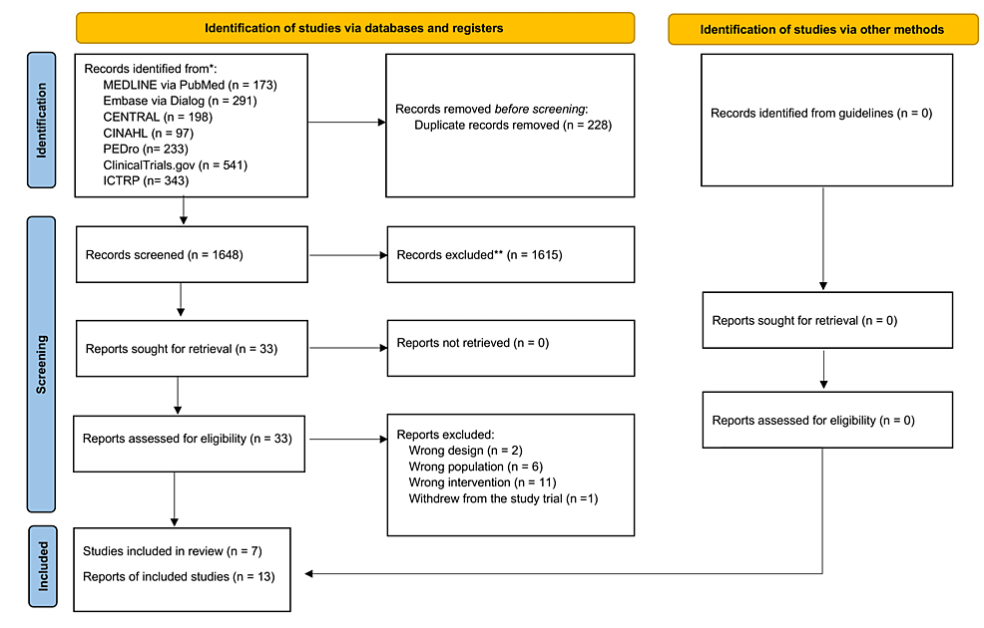
PRISMA flow diagram of the literature search results. PRISMA: Preferred Reporting Items for Systematic Reviews and Meta-Analyses.

We searched several databases and extracted 1,876 abstracts (Figure 1).

After removing duplicates, we finally screened 1,648 abstracts. During the full-text screening, six studies were excluded due to incorrect population [16-21], 11 due to incorrect intervention [22-32], two for incorrect design [33,34], and one for withdrawal [35]. Ultimately, we identified seven RCTs that met all the eligibility criteria (Figure 1, Table 1) [5, 6, 36-40].

**Table 1:** Characteristics of included studies. n (%), TME: total mesorectal excision; LAR: lower anterior resection; Ta-TME: transanal total mesorectal excision; PFMT: pelvic floor muscle training; NA: not applicable; LARS: low anterior resection syndrome; COREFO: Colorectal Functional Outcome Questionnaire; NRS: Numeric Rating Scale; SF-12: 12-Item Short Form Survey; FIQL: Fecal Incontinence Quality of Life Scale; QoL: quality of life; EORTC: European Organization for Research and Treatment of Cancer.

Author	Number of participants	Types of surgery	Stoma	Intervention	Controls	Outcomes
(Year)				(a: Timing of intervention initiation, b: duration, c: frequency, d: concomitant treatment)		
Asnong et al. [5] (2022)	104	TME	Yes 90 (87) No 14 (13)	a: One month after restoration of transit, b: 12 weeks, c: consisting of nine individual treatments (during the first six weeks once a week and three sessions over the last six weeks), d: assessment and evaluation of bowel symptoms with a stool diary, combined with patient education, biofeedback, electrical stimulation, and rectal balloon training	Did not receive any PFMT	LARS category LARS score COREFO questionnaire NRS regarding the subjective bother of bowel symptoms A stool diary (frequency of bowel movements, stool consistency, urgency/incontinence/soiling episodes, fragmentation of stool) SF-12 adverse events
Heijden et al. [6] (2022)	95	LAR	Yes 44 (46) Ileostomy 42 (95) Colostomy 2 (5)	a: Within three months after LAR or within six weeks after stoma closure, b: three months, c: NA, d: biofeedback, functional electrostimulation, rectal balloon training	Usual care (use of bulking agents, advice on lifestyle, fluid intake, use of fibers, diet, toilet posture)	Wexner score LARS score FIQL EORTC colorectal-specific QoL questionnaire (EORTC-QLQ-CR29) Safety Analysis
Lin et al. [36] (2016)	53	LAR	Yes 53 (100)	a: Day before discharge from the hospital, b: followed up for nine months, c: 20 contractions and relaxations 4 set/day, d: given exercise DVD	Pamphlet of post-surgical care	Wexner scale
Lau [37] (2010)	Protocol	LAR	NA	a: NA, b: NA, c: NA, d: NA	No intervention	Maximum squeeze pressure Bowel frequency FI score
Klarenbeek [38] (2015)	Protocol	Ta-TME	NA	a: NA, b: NA, c: NA, d: biofeedback, electrostimulation, balloon training	Regular treatment	Wexner score FIQL EORTC Colorectal QoL Questionnaire QLQ-CR38 Defecation diary LARS score Perioperative parameters Morbidity Mortality
Schiemer [39] (2017)	Protocol	TME	NA	a: NA, b: three months, c: 12 times, d: PFMT by specialized physiotherapists	No PFMT	LARS score QLQ-C30 score
Chuo [40] (2019)	Protocol	LAR	NA	a: NA b: NA c: NA d: biofeedback therapy	Blank control	LARS score QoL score

**Figure 2:**
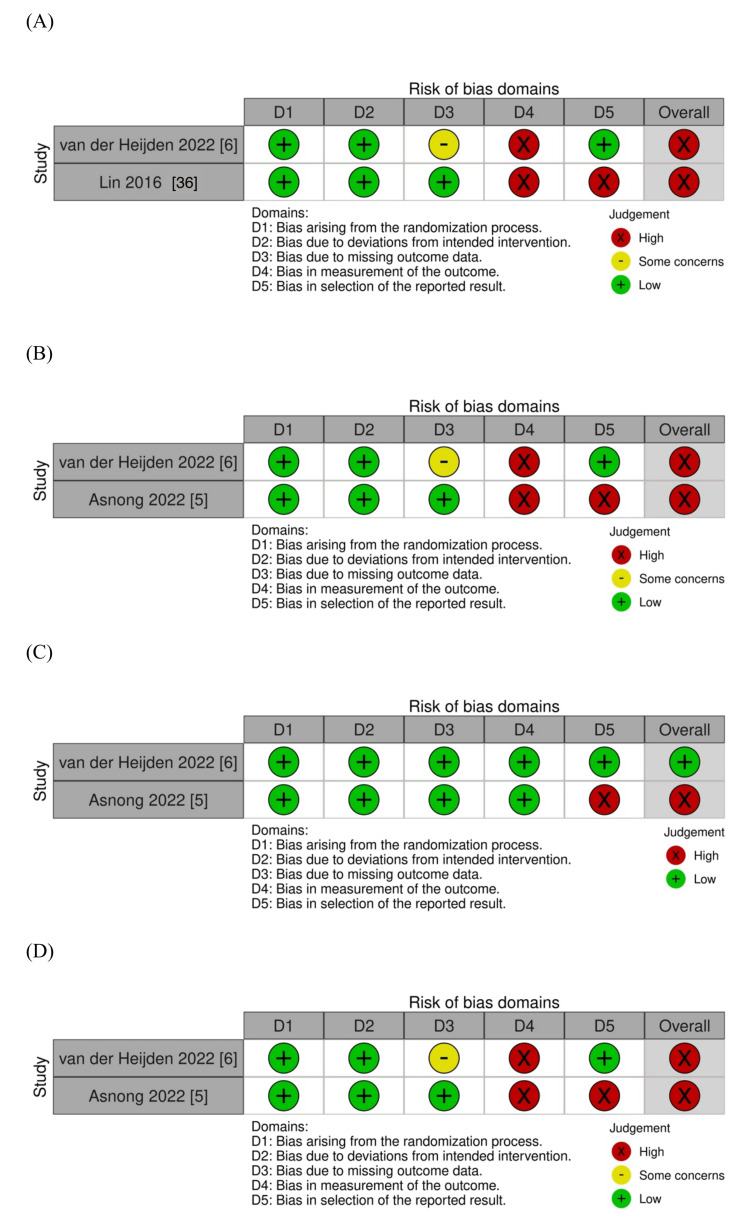
Risk of bias summary: (A) FI, (B) HR-QOL, (C) adverse events, and (D) bowel dysfunction after surgery. FI: fecal incontinence; HR-QOL: health-related quality of life.

The seven RCTs included 252 patients who underwent surgery for colorectal cancer and received PFMT postoperatively [5,6,36-40]. The types of surgery included total mesorectal excision (TME), lower anterior resection (LAR), and transanal total mesorectal excision (Ta-TME). The duration of the intervention ranged from three to nine months, and the frequency of the intervention ranged from once a week to every day. Patient education, biofeedback, electrical stimulation, and rectal balloon training were included as concomitant treatments for PFMT [5,6,38,40]. Most studies had a high overall risk of bias (Figure 2).

Primary Outcomes

**Figure 3:**
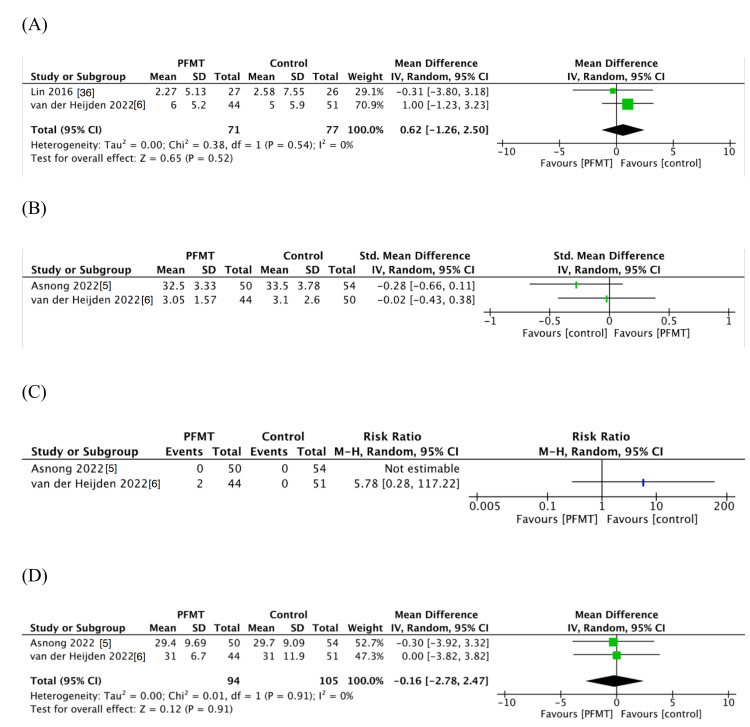
Forest plot of comparison: (A) FI, (B) HR-QOL, (C) adverse events, and (D) bowel dysfunction after surgery FI: fecal incontinence; HR-QOL: health-related quality of life.

The evidence suggested that PFMT in patients with colorectal cancer after surgery compared with controls had little to no difference in FI (two studies [6,36], 148 participants): mean difference (MD) 0.62 higher; 95% CI: -1.26 to 2.5; I^2^ = 0%; low certainty of evidence (CoE; Figures 2A, 3A; Table 2).

 

**Table 2:** Summary of findings: PFMT compared to control for health problem in colorectal cancer. CI: confidence interval; CoE: certainty of evidence; MD: mean difference; RR: risk ratio; SMD: standardized mean difference; PFMT: pelvic floor muscle training; HR-QOL: health-related quality of life; SF-12: 12-Item Short Form Survey; FIQL: Fecal Incontinence Quality of Life scale; LARS: Low anterior resection syndrome. ^a^Downgraded one level for limitations in the study design. ^b^Downgraded one level for imprecision (wide CI). ^c^Downgraded one level for inconsistency (heterogeneity of outcomes). ^d^Downgraded two level for imprecision (wide CI). ^*^The risk in the intervention group (and its 95% CI) is based on the assumed risk in the comparison group and the relative effect of the intervention (and its 95% CI).

Patient or population: health problem in colorectal cancer setting: any intervention: PFMT comparison: control						
Outcomes	Anticipated absolute effects^*^ (95% CI)		Relative effect (95% CI)	No. of participants (studies)	CoE (GRADE)	Comments
	Risk with placebo	Risk with PFMT				
FI (Wexner score)	-	MD 0.62 higher (1.26 lower to 2.5 higher)	-	148 (2 RCTs)	⨁⨁◯◯ Low^a,b^	
HR-QOL (SF-12, FIQL)	Not pooled	Not pooled	-	198 (2 RCTs)	⨁◯◯◯ Very Low^a,b,c ^	Only two studies reported HR-QOL data and the pooling of data was inappropriate due to heterogeneity of outcome measures. Therefore, individual study results are reported separately.
Adverse events	0 per 1,000	20 per 1,000 (0-70)	RR 5.78 (95% CI 0.28-117.22)	199 (2 RCTs)	⨁⨁◯◯ Low^a,b^	
Bowel dysfunction after surgery (LARS score)	-	MD 0.16 lower (2.78 lower to 2.47 higher)	-	199 (2 RCTs)	⨁◯◯◯ Very Low^a,d^	

The HR-QOL was measured in two studies: one study used the SF-12 and the other used the FIQL, with extremely low CoE (Figures 2B, 3B; Table 2) [5,6]. Therefore, we decided not to perform a meta-analysis using HR-QOL due to heterogeneity. In two studies, there was no trend toward a positive impact on HR-QOL [5,6].

Furthermore, PFMT results in little to no difference in adverse events (two studies [5,6], 199 participants): RR 5.78; 95% CI: 0.28-117.22; low CoE (Figures 2C, 3C; Table 2). No serious adverse events were reported [5,6], and two PFMT patients (anastomotic stricture, suboptimal laxative use) were referred to the outpatient clinic [6].

Secondary Outcome

The effect of PFMT in patients with colorectal cancer after surgery was uncertain on bowel dysfunction after surgery (two studies [5,6], 199 participants): SMD -0.16; 95% CI: -0.24- 2.47; I^2^ = 0%; extremely low CoE (Figures 2D, 3D; Table 2).

Discussion

To the best of our knowledge, this is the first SR and meta-analysis of RCTs on PFMT in patients following colorectal cancer surgery. We employed a rigorous methodology, adhering to a previously established written protocol based on the PRISMA 2020 statement, and performed an extensive search for supporting data. Our SR included seven trials with 252 patients. PFMT was utilized either alone [36] or in combination with biofeedback, electrical stimulation, and rectal balloon training [5,6]. The follow-up period extended from three to nine months from the initiation of the intervention. The results revealed that PFMT following surgery for colorectal cancer was unlikely to result in no or a trivial difference in FI and unlikely to lead to an increase in adverse events. Moreover, the results on HR-QOL in the reviewed studies were inconsistent.

However, our results indicate that there remain several unknown aspects of PFMT for postoperative patients with colorectal cancer. A previous SR that did not include RCTs showed that pelvic floor muscle rehabilitation after anterior resection of colorectal cancer is potentially beneficial [4]. Another SR has shown that PFMT is effective for bowel dysfunction; however, the study incorporated quasi-RCTs and RCTs [41]. In contrast, our review, which included only RCTs, revealed that despite several months of intervention, ranging from three to nine months, no clear benefits were achieved. Therefore, further RCTs and a longer intervention time are necessary to further validate these results.

Furthermore, PFMT in patients with colorectal cancer after surgery may result in little to no difference in adverse events. Treatment options for FI following colorectal cancer surgery include medications, fiber supplements, PFMT, and sacral nerve stimulator (SNS) implantation [42]. In our review, we found that initiating PFMT three months after low anterior resection (LAR) or four to six weeks after stoma closure did not lead to any serious adverse events. In our SR, 99 patients experienced adverse events, including anastomotic stenosis (one case, 1%) and inappropriate laxative use (one case, 1%). SNS was one of the treatments available for FI [42], and complications in 665 patients undergoing SNS included pain or local discomfort (37 cases, 6%), lead displacement or breakage (26 cases, 4%), and infection (22 cases, 3%) [43]. Therefore, PFMT should be considered for FI after colorectal cancer surgery due to its low adverse event rate.

Nonetheless, our study had limitations. First, the outcomes based on only RCTs in our SR and meta-analysis had a low to extremely low CoE, which indicates a need for higher-quality RCTs to provide more robust and reliable findings. Our SR identified a notable gap in the reporting of HR-QOL outcomes in studies involving PFMT in patients with colorectal cancer following surgery. Expert opinion on the core information set for colorectal cancer surgery highlights the importance of assessing the quality of life, including aspects such as physical and sexual functioning, FI, and urgency [44]. Furthermore, this finding underscores the necessity for conducting RCTs with HR-QOL outcomes that specifically focus on evaluating physical and sexual functioning, FI, and the sense of urgency in this patient population. In addition, our review also revealed a lack of clarity regarding the optimal duration and frequency of interventions. Therefore, to address this knowledge gap, we should specifically investigate the most effective and appropriate duration and frequency of PFMT intervention in postoperative patients with colorectal cancer and plan future RCTs that incorporate HR-QOL as an outcome.

## Conclusions

In conclusion, the findings of the SR suggest that PFMT has little to no trivial difference in improving FI and is associated with fewer adverse events. Our review emphasizes the importance of conducting high-quality RCTs to address the limitations and uncertainties regarding the optimal duration and frequency of PFMT interventions. HR-QOL should also be incorporated into the outcomes of RCTs. Furthermore, extending the duration of the PFMT intervention would be necessary to ensure its success.
